# Leigh-Like Syndrome With a Novel, Complex Phenotype Due to m.10191T>C in Mt-ND3

**DOI:** 10.7759/cureus.28986

**Published:** 2022-09-09

**Authors:** Shaundra M Newstead, Josef Finsterer

**Affiliations:** 1 Biochemistry, HeatSync Biochemistry Laboratory, Mesa, USA; 2 Neurology, Neurology and Neurophysiology Center, Vienna, AUT

**Keywords:** genetics, respiratory chain, mitochondrial disorder, mtdna, m.10191t>c

## Abstract

Leigh-like syndrome (LLS) due to the variant m.10191T>C in *ND3 *with a number of new phenotypic traits has not been published. In this case report, a 32-year-old woman diagnosed with Leigh-like syndrome presented with a complex novel, progressive, multisystem phenotype, manifesting in the brain (mild cognitive impairment, seizures, choreoathetosis, pseudotumor cerebri, hypersomnia, symmetric pallidal hypointensities, panda sign, calcifications, dysphagia), endocrine organs (empty sella syndrome, hypocorticism, hypoaldosteronism, hypogonadism), hematopoietic system (anemia, lymphocytosis), immune system (lymphocytosis, hypogammaglobulinemia), gut (reflux, diarrhea), kidneys (renal insufficiency, renal tubular acidosis, nephrolithiasis), muscles (myopathy, exercise intolerance, easy fatigability), peripheral nerves (small fiber neuropathy, dysautonomia), connective tissue (hyperlaxity of joints, bruising), and bones (scoliosis, Chiari malformation). A genetic workup revealed the known pathogenic variant m.10191T>C in *ND3*, which was also carried by the patient’s mother. This case demonstrates that the m.10191T>C variant in *ND3* can phenotypically manifest with multisystem disease and that this disease is responsive to symptomatic treatment and application of additional compounds.

## Introduction

Leigh syndrome is the most common mitochondrial disorder (MID) in pediatric patients, with an estimated incidence of 1 in 40,000 live births [1]. Leigh syndrome is characterized by progressive loss of mental and movement abilities (psychomotor regression) resulting in feeding problems, respiratory failure, and bilateral basal ganglia lesions on imaging. In addition to classical Leigh syndrome, there are a number of respiratory chain defects that also manifest with basal ganglia defects but with additional widespread multisystem involvement including the peripheral nerves, muscles, endocrine system, heart, liver, kidneys, and hematopoietic system (Leigh-like syndrome [LLS]) [2]. The onset of the disorder can be in early infancy or adulthood. Some patients with early-onset Leigh syndrome survive into adulthood. Currently, no successful treatment for Leigh syndrome exists, but there are many promising candidates. The prognosis is generally poor for classical Leigh syndrome and LLS, but fair for their adult-onset forms and patients who survive into adulthood. Leigh syndrome and LLS, similar to many other MIDs, have a heterogeneous genetic background and manifest with heterogeneous phenotypic expression and lifespan [2]. This case report seeks to introduce Leigh syndrome and LLS into the physician’s clinical index of suspicion, even if the patient is older than the typical age of presentation or has an atypical phenotype [3-13].

## Case presentation

The patient was a 32-year-old woman diagnosed with LLS with a height and weight of 170 cm and 120 kg, respectively, born to non-consanguineous parents, with the following history (Table 1): severe new-born jaundice, delayed speech development (stumbling until age 2), joint hypermobility from age 3 and recurrent sprains, dislocations, and easy bruising; exercise intolerance, reduced endurance, and exercise-induced muscle burning from age 5 which improves with rest, cool rags, and sugar, depression from age 5 being treated with antidepressants from age 12, micropsia from age 6, recurrent severe diarrhea from age 6 which did not stop earlier than after administration of ubiquinol at age 26, a first syncope of unknown etiology at age 7 which progressed to several syncopes per day around age 14 attributed to suspected orthostatic hypotension, migraine without aura manifesting with unilateral throbbing pain in the right middle cerebral artery starting at age 7 which responds to sumatriptan and indomethacin, and focal or generalized seizures starting with staring spells, but also tonic-clonic seizures with a frequency of several seizures per year from age 7.

**Table 1:** Evolution of clinical manifestations over years and comparison with previously reported carriers of the m.10191T>C variant Ic: index case, LP: lumbar puncture, MSSA: methicillin-susceptible staphylococcus aureus, Na: not applicable, ur: unreported

Manifestation	Onset (y)	Intervention	Outcome	IC	Reference
Newborn jaundice	0	none	resolved	x	[ur]
Delayed speech development	1	none	resolved	x	[ur]
Joint hypermotility	3	none	persisting	x	[ur]
Exercise intolerance	5	aerobic training, short rest, supplements	progressing	x	[ur]
Exercise-induced muscle burn	5	analgesics, short rest	progressing	x	[ur]
Depression	5	antidepressants	persisting	x	[ur]
Micropsia	6	none	persisting	x	[ur]
Diarrhoea	6	ubiquinol	resolved	x	[ur]
Syncope	7	midodrine, aerobic training	improved	x	[4]
Migraine	7	ubiquinol,sumatriptan	resolved	x	[14]
Focal and generalised seizures	7	gabapentin	persisting	x	[3,14]
Exercise-induced fatigue	11	rest, anti-inflammtories	persisting	x	[ur]
Hypogonadism	12	none	persisting	x	[ur]
Mild cognitive impairment	14	intell. activity, amantadin	progressing	x	[14-17]
Derealisation	14	warm light	recurring	x	[ur]
Pseudotunor cerebri, papilledema	14	azetazolamide, LP, dexamethasone at age 21	resolved	x	[ur]
Impaired fatty acid oxidation	14	fenofibrate	persisting	x	[ur]
Hypersomnia	14	none	persisting	x	[ur]
Kidney stones	18	surgery	4 relapses	x	[ur]
Craniocervical instability	18	none	persisting	x	[ur]
Chiari malformation	18	none	persisting	x	[ur]
Lancinating nerve pain	19	gabapentin	resolved	x	[ur]
Arrhythmias, bradycardia	19	none	persisting	x	[12]
Drops in blood pressure	19	midodrine	improved	x	[ur]
Central hypoventilation	19,23,26	aerobic training, ubiquinol	persisting	x	[ur]
Microarrousals on sleep studies	19,23,26	none	persisting	x	[ur]
Somniloquy	19,23,26	none	persisting	x	[ur]
Exercise tachycardia, dyspnoea	20	none	persisting	x	[ur]
Renal insufficiency	20	fluid substitution	persisting	x	[ur]
Lymphadenopathy	21	none	persisting	x	[ur]
Recurrent central apnoe	21	none	persisting	x	[ur]
Lactic acidosis	22	antioxidants, pyruvate	persisting	x	[ur]
Adrenal insufficiency	22	coritsol	persisting	x	[ur]
Steroid myopathy	22	withdrawal	improved	x	[ur]
Exercise-induced muscle cramping	22	rest	persisting	x	[ur]
Choreo-athetosis	23	amantadin	persisting	x	[ur]
Orthostatic hypotension (tilt test)	23	midodrine	persisting	x	[ur]
Small fiber neuropathy	23	none	persisting	x	[ur]
Cerebellar ataxia	25	none	persisiting	x	[10,14]
Left eye ptosis	25	none	persisting	x	[ur]
Vertical diplopia	25	none	persisting	x	[ur]
Horizontal left nystagmus	25	none	persisting	x	[ur]
Hypoaldosteronism	25	none	persisting	x	[ur]
Pancytopenia	25	splenectomy	resolved	x	[ur]
Haemolytic anemia	25	splenectomy	resolved	x	[ur]
Poikilocytosis	25	none	persisting	x	[ur]
Adverse reaction to propofol	26	avoidance	resolution	x	[ur]
Dextro-scoliosis	26	none	persisting	x	[ur]
Renal tubular acidosis	26	none	persisting	x	[ur]
Sepsis (MSSA)	26	antibiotics	resolved	x	[ur]
Acute respiratory distress (syndrome	26	oxygen, antibiotics	resolved	x	[ur]
Hypertriglyceridemia	26	fenofibrate	improved	x	[ur]
Respiratory alkalosis	27	none	persisting	x	[ur]
Dysphagia, respiratory acidosis	27	none	persisting	x	[13]
B-cell lymphocytosis	29	none	persisting	x	[ur]
Hypogammaglobulinemia	30	none	persisting	x	[ur]
Empty sella	30	none	persisting	x	[ur]
Infant lethality	na	none	na	no	[5]
Developmental delay	na	none	na	no	[3]
Homonymous hemianopia, anopia	na	none	na	no	[9,16]
Myoclonic epilepsy	na	antiseizure drugs	na	no	[9]
Myocloni	na	antiseizure drugs	na	no	[4,10,16]
Optic atrophy	na	none	na	no	[14,15]
Cerebellar ataxia	na	none	na	no	[14]
Large fiber neuropathy	na	none	na	no	[14]
Spasticity	na	none	na	no	[14]
Dystonia	na	none	na	no	[3]
Visual impairment	na	none	na	no	[14]
Ophthalmoparesis	na	none	na	no	[14,18]
Myopathy	na	none	na	no	[4,14]
Cardiac arrest, cardiomyopathy	na	none	na	no	[13,15]
Short stature	birth	none	na	no	[16]
Nystagmus	na	none	na	no	[16]
Hearing loss	na	none	na	no	[18]
Intermittent tremor	na	none	na	no	[19]
Vomiting	na	symptomatic	na	no	[13]
Hypothermia	na	symptomatic	na	no	[13]
Micrognathia	na	none	na	no	[12]
Pes equinovarus	na	none	na	no	[12]
Bulbar signs, apnea, bradypnoea	na	none	na	no	[7,8,11]
Stroke-like episodes	na	L-arginine	na	no	[9,18,20]
Poor feeding	na	none	na	no	[11,13]
Macrocephaly	na	none	na	no	[8]

The history continues with exercise-induced fatigue from age 11, hypogonadism recognized at age 12, pseudotumor cerebri with papilledema diagnosed at age 14 with acetazolamide, methazolamide, repeated lumbar punctures, and dexamethasone treatment beginning at age 21, hypersomnia from age 14, mild cognitive impairment and derealization from age 14 progressing to severe executive dysfunction and language-finding difficulties on repeated neuropsychological testing, kidney stones first diagnosed at age 18 and four times thereafter, permanent lymphocytosis from age 19, lancinating nerve pain from age 19 which responds to gabapentin, episodes of supraventricular tachycardia, premature ventricular beats, and exercise-induced sinus tachycardia on Holter monitoring at age 19, sleep studies at ages 19, 23, and 26 revealing recurrent central hypoventilation, which improved with aerobic training and ubiquinol; mild renal insufficiency from age 20; MID suspected by name at age 20;

The medical history goes on with intermittent adrenal insufficiency (hypocorticism) successfully treated with hydrocortisone from age 21, recurrent episodes of central apnea lasting up to 30 seconds from age 21, intermittently elevated serum lactate from age 22, and a lactate/pyruvate ratio reaching 28, choreoathetotic hyperkinesia starting at age 23, severe orthostatic hypotension on tilt table testing at age 23, small fiber neuropathy diagnosed from a skin biopsy at age 23, hypoaldosteronism diagnosed at age 25, hemolytic anemia (low erythrocyte count, low hematocrit, high red cell distribution width, elevated lactate dehydrogenase-A, low haptoglobin, low reticulocytes, hypersplenism) from age 25 which was treated with a splenectomy at age 26 under propofol and isoflurane, general anesthesia for splenectomy complicated by a 12-h delay of awakening and severe thrashing ballism, 16° Cobb angle dextro-scoliosis first diagnosed at age 26, hyperlipidemia from age 26, empty sella syndrome diagnosed at age 30, and hypo-gammaglobulinemia first noted at age 30. There were also chronically elevated inflammatory markers, including an erythrocyte sedimentation rate of 88 (not during hemolysis, when it was higher), a C-reactive protein level of 110 mg/L, recurring retroperitoneal, abdominal, thoracic, and cervical lymphadenopathy, very high CD19 counts, mild hypogammaglobulinemia, and borderline-low immunoglobulin M. However, extensive investigations into autoimmune diseases were uninformative.

**Figure 1:**
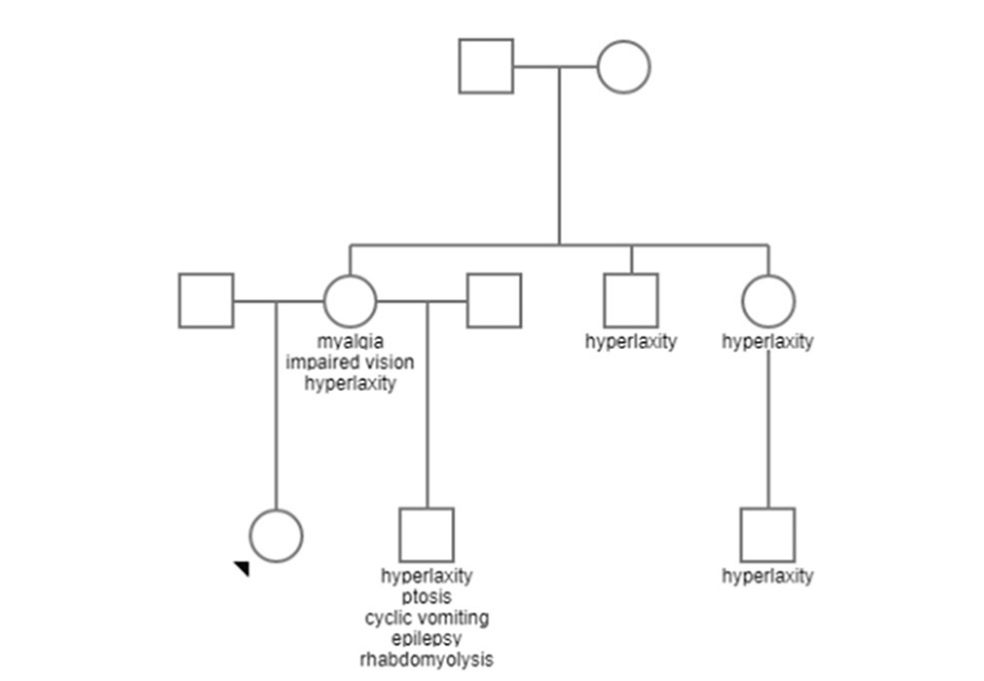
Pedigree of the index patient

The patient’s mother suffered from myalgias, hyperlaxity, and bilateral progressive visual impairment. The patient’s half-brother (same mother) had moderate left ptosis, cyclic vomiting syndrome, epilepsy, and rhabdomyolysis (Figure 1).

**Figure 2:**
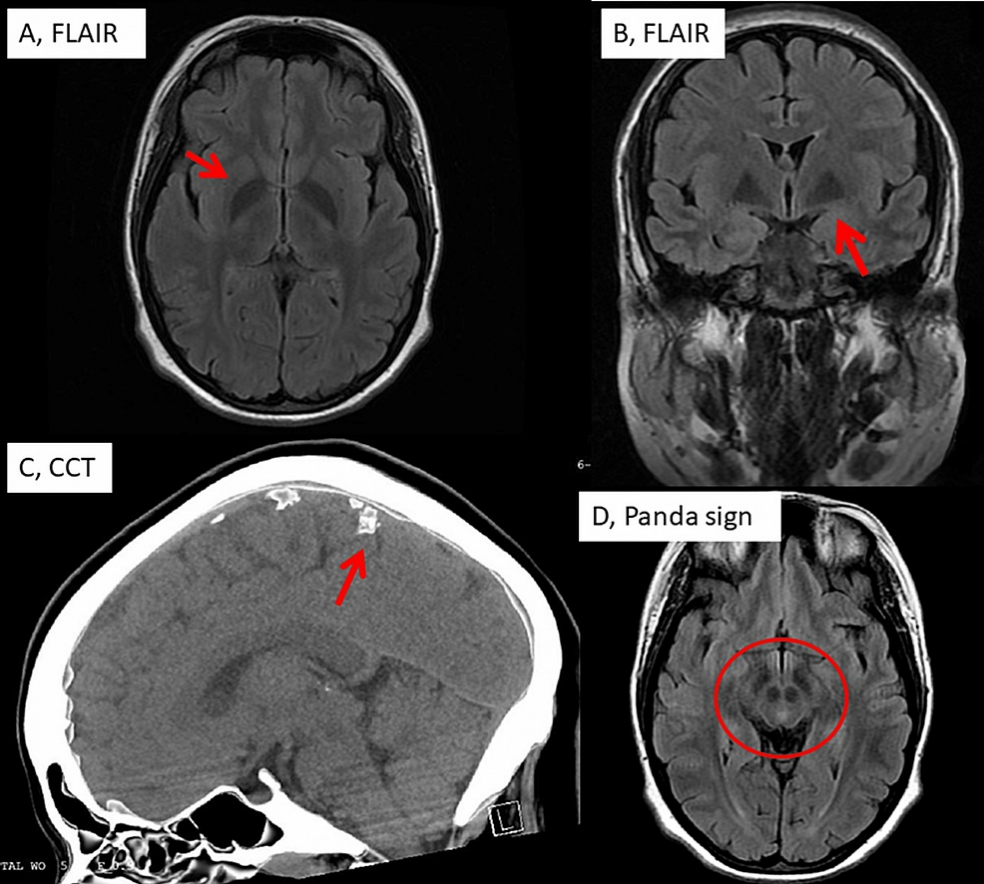
Magnetic resonance imaging (axial and coronary FLAIR images) showing hypointensity of the globus pallidus bilaterally (A, B). FLAIR images of the midbrain showing a distinct Panda sign (D). Cerebral CT of the brain (sagittal plane) showing abnormal calcifications in the subarachnoid space CCT: cerebral CT, FLAIR: fluid attenuated inversion recovery

A clinical neurological exam revealed left ptosis, vertical diplopia, horizontal nystagmus, occasional choreoathetosis, and ataxia on tandem gait. Nerve conduction studies and needle electromyography were uninformative. Cerebral CT revealed abnormal calcifications (Figure 2). Cerebral MRI demonstrated mild periventricular white matter hyperintensities, bilateral T2 and FLAIR hypointense lesions within the globus pallidus and midbrain, and a “face of the giant panda” sign (Figure 1). No lactate peak was seen in magnetic resonance spectroscopy. Creatine kinase level was normal. There was mild renal insufficiency and mild hypertriglyceridemia. Lactate was slightly elevated but pyruvate was normal at rest. The lactate pyruvate ratio was 28 but decreased with aerobic training and medication. Serum levels of cortisol, adreno-corticotropic hormone, epinephrine, and aldosterone were lower than normal. The urine amino acid profile demonstrated very low organic acids (glycolic, 3-hydroxypropionic, 2-hydroxy-isovaleric, succinic, methyl-succinic, glutaric, malic, 3-hydroxy-adipic, adipic, 5-oxoproline, citric, isocitric), which is why renal tubular acidosis was diagnosed. A 6-minute walking test (6MWT) of arterial blood gases revealed hypoxia (38 mmHg) with uncompensated lactic acidosis immediately after exercise and uncompensated respiratory alkalosis 5 minutes after exercise. A second 6MWT 20 minutes later revealed post-exercise lactic acidosis with hyperoxia (excess of oxygen). VO_2_ max testing demonstrated 14 mL O_2_/kg/min, which increased to 16 mL O_2_/kg/min with aerobic training. Neuropsychological testing demonstrated severe executive dysfunction, language-finding difficulties, and reduced processing speed which worsened on repeat testing.

**Figure 3:**
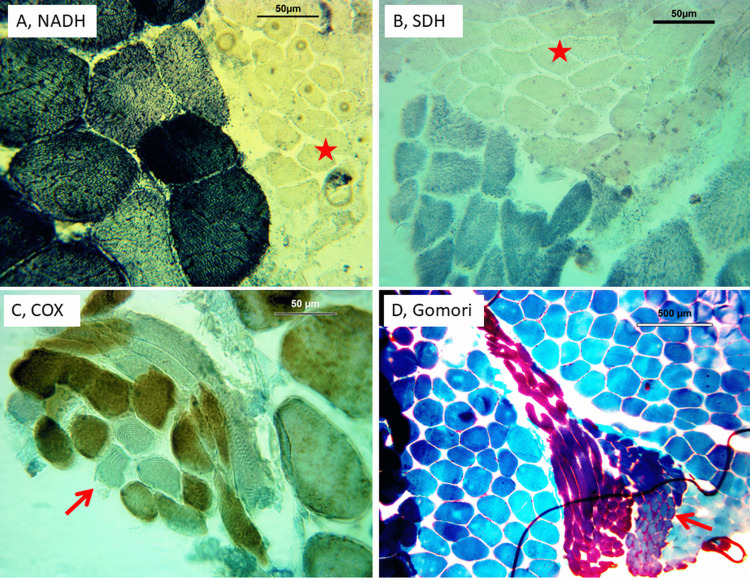
Muscle biopsy from the right rectus femoris muscle showing absence of NADH staining in atrophic fibers (A, star), atrophic fibers that are SDH negative or SDH positive (B, star), several COX negative muscle fibers (C, arrow), and atrophic ragged-red fibers on Gomori trichrome staining (D, arrow) COX: cytochrome-C oxidase, NADH: nicotine adenine, dinucleotide, SDH: succinate dehydrogenase

Muscle biopsy revealed >10% ragged-red fibers with corresponding ragged-blue fibers on succinate dehydrogenase staining, subsarcolemmal deposits on succinate-dehydrogenase staining, 2% cytochrome-C oxidase-negative fibers which was at a frequency much greater than would be expected in a patient of this age-and denervation with fiber-type grouping (Figure 3).

A genetic workup at age 23 revealed the mtDNA variant m.10191T>C in MT-ND3 (p.Ser45Pro) and the variant m.12770A>G in MT-ND5 in the buccal mucosa cells. The variant was confirmed two more times by whole exome sequencing and mtDNA sequencing. Heteroplasmy rates were not determined. The mother also tested positive for the m.10191T>C variant.

At the time of the study, the index patient was on polypharmacy with 46 compounds (Table 2). Despite being a triathlete and competitive para-swimmer, and eating <1000 kcal/d, the patient had difficulty losing weight. The patient is currently able to sufficiently perform all activities of daily living. Despite mild progression, the prognosis has to be assessed as good.

**Table 2:** Current medication the patient is taking every day GABA: gamma-aminobutyric acid, SAMe: S-adenosyl-L-methionine

Drug	Morning	Afternoon	Night
Amantadine	0	0	100 mg
Berberine	0	0	1200 mg
Biotin	10000 mg	10000 mg	0
Acetyl-L-carnitine	1000 mg	1000 mg	1000 mg
Creatine-hydrochloride	1000 mg	1000 mg	0
N-acetyl-cysteine	600 mg	0	0
Diclofenac	75 mg	0	0
Fenofibrate	200 mg	0	200 mg
Folic acid	1000 mg	0	0
Dimethyl-fumarate	120 mg	0	0
GABA	0	0	750 mg
Gabapentin	0	0	300 mg
Glutamine	0	0	1000 mg
Glycine	0	0	2000 mg
Trimethyl-glycine	0	0	750 mg
Magnesium glycinate	0	0	1000 mg
Alpha lipoic acid	700 mg	100 mg	0
Lutein	20 mg	0	0
Manganese gluconate	50 mg	0	0
Melatonin	0	0	10 mg/d
Midodrine	10 mg	0	0
Nicotinamide	300 mg	300 mg	0
Omega-3 oil	4000 mg	0	0
Omeprazole	40 mg	0	0
Pantothenate	0	500 mg	0
Pentoxifylline	400 mg	0	0
Triphenyl-phosphonium ubiqutinol	5 mg	0	0
Pyrroloquinoline quinone	20 mg	20 mg	0
Sodium pyruvate	2000 mg	2000 mg	1000 mg
Riboflavin	0	400 mg	0
D-ribose	4000 mg	1000 mg	0
SAMe	400 mg	400 mg	0
Selenium	200 mg	200 mg	0
Sertraline	100 mg	0	0
Diethyl-succinate	0	0	0.3 mL
Taurine	500 mg	0	0
Theanine	0	500 mg	0
Thiamine	0	500 mg	0
L-tryptophan	0	0	500 mg
Ubiquinol	600 mg	300mg	0
Triphenyl-phosphonium ubiquinol	5 mg	0	0
Vitamin-A	10000 IU	0	0
Vitamin-C	0	0	100 mg
Vitamin-D	5000 IU	0	0
Vitamin-E	400 IU	400 IU	0
Zinc-gluconate	50 mg	0	0

## Discussion

The index patient was interesting in several respects. First, the patient was diagnosed with progressive LLS which began in childhood and continued into adulthood. Second, the patient showed previously undescribed phenotypic characteristics of a multisystem MID due to the m.10191T>C variant. Accordingly, LLS manifested not only in the brain (mild cognitive impairment, seizures, choreoathetosis, pseudotumor cerebri, hypersomnia, symmetric pallidal hypointensities, panda sign, calcifications, empty sella, dysphagia) but also in the endocrine organs (hypocorticism, hypoaldosteronism, empty sella, hypogonadism), hematopoietic system (anemia), cellular immune system (lymphocytosis, hypogammaglobulinemia, high CD19 cells, B-cell lymphocytosis), gut (reflux and diarrhea), kidneys (renal insufficiency, renal tubular acidosis, nephrolithiasis), muscles (ptosis, myopathy, hyperoxia), peripheral nerves (small fibre neuropathy, dysautonomia), connective tissue (easy bruising, impaired wound healing, hyperlaxity of joints), and bones (scoliosis, Chiari malformation). Third, the patient was in extreme polypharmacy mainly due to experiences with self-medication.

Previously reported phenotypic manifestations of m.10191T>C carriers were comprehensively reviewed in a report by Li et al., in which 28 patients were discussed [4]. Phenotypic manifestations of the m.10191T>C variant reported in these 28 patients included cognitive impairment, migraine, seizures, anopia or hemianopia, myocloni, myoclonic epilepsy, stroke-like episodes, ataxia, optic atrophy, large fiber neuropathy, spasticity, nystagmus, tremor, myopathy including ptosis and ophthalmoparesis, hearing loss, cardiac involvement including bradycardia, hypothermia, vomiting, micrognathia, pes equinovarus, and short stature (Table 1) [4]. Of these manifestations, only cognitive impairment, epilepsy, delayed speech development, and migraine were also found in the index patient. Another study investigated seven patients carrying the m.10191T>C variant; interestingly, none presented with central nervous system involvement, whereas four presented with myopathy, one with respiratory failure, six with cardiac involvement, one with gastrointestinal disease, three with endocrinopathy, four with renal disease, three with ophthalmologic impairment, six with hypoacusis, and five with bone abnormalities [3]. Another patient, a 24-year-old woman with the m.10191T>C variant, only started presenting clinical manifestations at the age of 18 when she developed myocloni and seizures [4]. During the course of the disease, she also developed mild quadriparesis and generalized muscle wasting due to myopathy [4]. Therefore, the patient’s phenotype differed in some aspects from other reports of patients with the m.10191T>C variant (Table 1).

The high number of unusual phenotypic manifestations in the index patient as compared to previous reports could be explained by variants in genes other than ND3. Whether the m.12770A>G variant in MT-ND5 contributed to the phenotypic heterogeneity remains speculative, this variant has been previously associated with mitochondrial encephalopathy, lactic acidosis, and stroke-like episode syndrome. An argument against mutations in nuclear mitochondrial genes contributing to the phenotypic heterogeneity is that none were present in whole exome sequencing. The multisystem nature of LLS is a strong argument for its diagnosis, as is the striking cerebral imaging. In this case report, cerebral imaging was not only striking for bilateral pallidal hypointensities but also for the face of the giant panda sign, or the “panda sign,” which refers to the appearance of the midbrain when the red nucleus and substantia nigra are surrounded by a high T2 signal in the tegmentum. The panda sign is classically seen in Wilson’s disease, although a similar appearance is seen whenever the white matter in the region is diffusely abnormal. In addition to Wilson’s disease, the panda sign has been described in Japanese encephalitis, rabies, sarcoidosis, acute disseminated encephalitis, toxic leukoencephalopathy, Leigh syndrome, and LLS. With the exception of LLS, all other differential diagnoses with the panda sign were ruled out in the index patient based on clinical presentation, disease course, laboratory findings, and imaging.

Interestingly, the patient did not require anti-seizure drugs other than gabapentin. This may be because she was also taking fenofibrate, which is known to increase ketone bodies. Ketone bodies are known to have an anticonvulsant effect, particularly in patients with a MID (ketogenic diet).

The high CD19 count, mild hypogammaglobulinemia, and borderline-low immunoglobulin M may have been due to the heavy reliance of B cells on oxidative phosphorylation compared to T cells. C10orf2 variants have been reported to affect immune cells in MID patients. In addition, systemic inflammation has been documented in NDUFS4 knockout mice.

The present study was limited in that the heteroplasmy rate was not determined, and no biochemical studies were performed to assess respiratory chain functions. However, the m.10191T>C variant showed poor correlations with heteroplasmy, symptoms, and lifespan.

## Conclusions

This case demonstrates that the m.10191T>C variant in ND3 can phenotypically manifest with a multisystem disease affecting the brain, muscles, nerves, endocrine system, intestines, kidneys, blood cells, immune cells, bones, and joints, which responds to polypharmacy. In LLS due to an mtDNA defect, symptomatic treatment and the use of complementary preparations may be beneficial. This case expands the phenotypic spectrum of the m.10191T>C variant and provides new perspectives for its treatment.
